# Towards the Bisbenzothienocarbazole Core: A Route of Sulfurated Carbazole Derivatives with Assorted Optoelectronic Properties and Applications

**DOI:** 10.3390/ma14133487

**Published:** 2021-06-23

**Authors:** Roger Bujaldón, Joaquim Puigdollers, Dolores Velasco

**Affiliations:** 1 Grup de Materials Orgànics, Institut de Nanociència i Nanotecnologia (IN2UB), Departament de Química Inorgànica i Orgànica, Secció de Química Orgànica, Universitat de Barcelona, Martí i Franquès, 1, E-08028 Barcelona, Spain; rr.bujaldon@ub.edu; 2 Departament d’Enginyeria Electrònica, Universitat Politècnica de Catalunya, Jordi Girona, 1-3, E-08034 Barcelona, Spain; joaquim.puigdollers@upc.edu

**Keywords:** blue emission, carbazole, fluorophores, ladder-type molecules, optoelectronics, organic chemistry, organic semiconductors, sulfur, thin-film transistors

## Abstract

Ladder-type molecules, which possess an extended aromatic backbone, are particularly sought within the optoelectronic field. In view of the potential of the 14*H*-bis[1]benzothieno[3,2-*b*:2’,3’-*h*]carbazole core as a p-type semiconductor, herein we studied a set of two derivatives featuring a different alkylation patterning. The followed synthetic route, involving various sulfurated carbazole-based molecules, also resulted in a source of fluorophores with different emitting behaviors. Surprisingly, the sulfoxide-containing fluorophores substantially increased their blue fluorescence with respect to the nearly non-emitting sulfur counterparts. On this basis, we could shed light on the relationship between their chemical structure and their emission as an approach for future applications. Considering the performance in organic thin-film transistors, both bisbenzothienocarbazole derivatives displayed p-type characteristics, with hole mobility values up to 1.1 × 10^−3^ cm^2^ V^−1^ s^−1^ and considerable air stability. Moreover, the role of the structural design has been correlated with the device performance by means of X-ray analysis and the elucidation of the corresponding single crystal structures.

## 1. Introduction

Organic-based semiconductors have steadily aroused the interest of the scientific community during the last decades, spreading and proving their functionality in devices such as organic thin-film transistors (OTFTs), organic light-emitting diodes (OLEDs) and organic solar cells (OSCs) [1,2,3]. Indeed, organic semiconductors claim coveted properties that provide numerous advantages over their traditional inorganic-based counterparts. The fact that their electronic characteristics can be easily modulated synthetically and the feasibility of fabricating flexible displays with large areas at a lower cost are just a few of them [1,4,5,6].

In order to pave the way towards high performing OTFTs, the molecular architecture of the semiconductor requires an accurate and rational design. It should afford the material the appropriate intrinsic electronic properties as well as ensuring an efficient charge transport between neighboring molecules [4,7,8,9]. Ladder-type constructions, with particularly planar aromatic cores that promote extended intermolecular π–π stacking interactions, are well-known examples [10,11,12]. Pentacene is probably one of the best representatives of this type of compound. Despite its disadvantageous lack of stability, it still stands among the top organic semiconductors, with reported charge mobility values up to 35 cm^2^ V^−1^ s^−1^ determined by space-charge-limited current measurements [13,14]. The inclusion of heterocycles into the pentacene backbone has been a frequently exploited strategy in the search for novel materials with enhanced stability and performance. Notably, many derivatives containing or combining thiophene, pyrrole or even more extended cores such as [1]benzothieno[3,2-*b*][1]benzothiophene (BTBT) have achieved this goal [15,16,17,18,19,20]. Carbazole as a synthetic building block also represents an important source of organic molecules with assorted applications within the optoelectronic field. In fact, it does not only stand as a promising scaffold for new organic semiconductors, but also displays outstanding photophysical properties [9,20,21,22,23,24].

**Figure 1:**
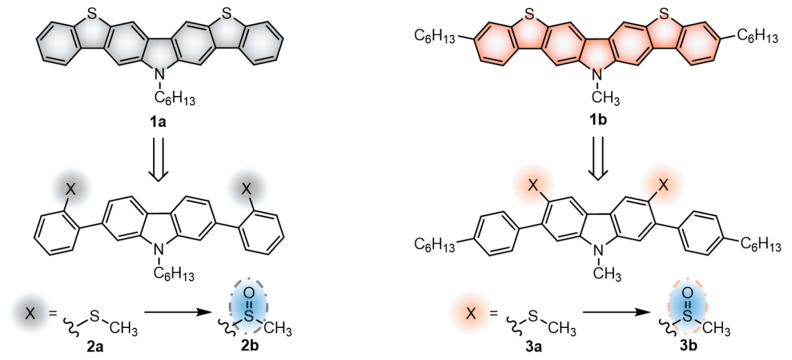
Schematic representation of the retrosynthetic approach towards compounds **1a**–**b** and their respective synthetic precursors **2a**–**b** and **3a**–**b**.

The herein studied 14*H*-bis[1]benzothieno[3,2-*b*:2’,3’-*h*]carbazole core, being a conjugated heteroacene fusing both carbazole and thiophene moieties, has the potential to fulfill this role. Albeit the promising performance of similar constructions in OTFTs and the efforts employed in developing a suitable synthetic route [25,26], to the best of our knowledge there are no reported results of devices integrating this particular core. In this work, two derivatives of the bisbenzothienocarbazole core featuring a different alkylation patterning have been integrated in OTFTs (Figure 1). In addition to enhancing the solubility in common organic solvents, the presence of alkyl chains plays a key role in modulating the intermolecular interactions, the disposition in the solid state and therefore the performance of the final device [8,27,28,29]. Specifically, the derivative **1a** has a hexyl chain attached to the central nitrogen, whereas **1b** possesses an *N*-methyl, the shortest alkyl chain, with two peripheral hexyl chains to confer a more elongated architecture. Keeping in mind that the carbazole ring also exhibits interesting photophysical properties, we also analyzed the emission of the sulfurated synthetic precursors necessary to obtain compounds **1a**–**b**. The retrospective synthetic route towards **1a**–**b** is shown in Figure 1. This set of compounds displays sulfur in two different oxidation states: the intermediates **2a** and **3a**, bearing two methylthio groups, and their oxidized methylsulfinyl counterparts **2b** and **3b**. It should be noted that the oxidation of the methylthio unit implies the conversion from an electron-donating to an electron-withdrawing group. The associated electrochemical modulation is therefore prone to affecting their photoluminescence. Indeed, the switch from methylsulfinyl to methylthio groups in various fluorophores has recently been reported as a tool for biological imaging probes [30,31]. In our case, the location of this substituent varies between structures **2** and **3**, providing the opportunity to shed light on its effect on the photoluminescence and to envision new applications in diverse fields.

## 2. Materials and Methods

### 2.1. Synthesis and Characterization

The commercially available chemicals were employed as received. Concerning the solvents, they were dried and degassed using standard procedures. Specifically, tetrahydrofuran (THF) was dried by distillation from sodium and benzophenone, whereas dichloromethane and acetonitrile from CaH_2_. Anhydrous CHCl_3_ (Sigma-Aldrich, St. Louis, MO, USA) was kept under nitrogen atmosphere. Flash chromatography was performed over commercial silica gel (VWR, 40–63 µm).

#### 2.1.1. Synthesis of 2,7-bis(2-methylthiophenyl)-9-hexyl-9*H*-carbazole (**2a**)

2,7-Dibromo-9-hexyl-9*H*-carbazole **4a** (1.15 g, 2.80 mmol), 2-methylthiophenylboronic acid (1.22 g, 7.28 mmol), Pd(PPh_3_)_4_ (0.161 g, 0.139 mmol) and K_2_CO_3_ (2.33 g, 16.8 mmol) were placed in a round-bottom flask. Then, the system was purged with nitrogen. A previously purged mixture of THF and water (6:1 *v*/*v*, 58 mL) was added to the flask and stirred at reflux overnight. The reaction mixture was extracted with CH_2_Cl_2_, dried over MgSO_4_ and the solvent was removed under reduced pressure. The crude was purified by flash column chromatography using a mixture of hexane and dichloromethane (20:1 *v*/*v*) as eluent. Compound **2a** was obtained as a white solid in a yield of 80% (1.11 g, 2.25 mmol). ^1^H NMR (400 MHz, CDCl_3_) δ (ppm): 8.14 (d, *J* = 8.0 Hz, 2H), 7.47 (d, *J* = 8.0 Hz, 2H), 7.40–7.31 (m, 6H), 7.29–7.23 (m, 4H), 4.30 (t, *J* = 7.3 Hz, 2H), 2.37 (s, 6H), 1.94–1.83 (m, 2H), 1.45–1.20 (m, 6H), 0.84 (t, *J* = 7.1 Hz, 3H). ^13^C NMR (100 MHz, CDCl_3_) δ (ppm): 141.9, 140.8, 138.2, 137.7, 130.5, 127.9, 125.3, 124.8, 122.2, 120.5, 120.2, 109.9, 43.4, 31.8, 29.3, 27.2, 22.7, 16.1, 14.2.

#### 2.1.2. Synthesis of 2,7-bis(2-methylsulfinylphenyl)-9-hexyl-9*H*-carbazole (**2b**)

**2a** (602 mg, 1.21 mmol) was dissolved in glacial acetic acid (40 mL) and cooled at 0 °C. Hydrogen peroxide (35%, 218 µL, 2.55 mmol) was added carefully and the mixture was stirred overnight at room temperature. After, the solvents were removed under reduced pressure. The crude was purified by flash column chromatography using a mixture of hexane and ethyl acetate (1:1 *v*/*v*) as eluent. Compound **2b** was obtained as a pale yellow solid in a yield of 94% (600 mg, 1.14 mmol). ^1^H NMR (400 MHz, CDCl_3_) δ (ppm): 8.19 (d, *J* = 7.9, 2H), 8.17 (d, *J* = 7.9, 2H), 7.67 (td, *J* = 7.6, 1.4 Hz, 2H), 7.60 (td, *J* = 7.4, 1.4 Hz, 2H), 7.25 (dd, *J* = 7.4, 1.4 Hz, 2H), 7.46 (s, 2H), 7.25 (dd, *J* = 7.9, 1.4 Hz, 2H), 4.32 (t, *J* = 7.2, 2H), 2.34 (s, 6H), 1.87 (m, 2H), 1.45–1.20 (m, 6H), 0.83 (t, *J* = 7.1 Hz, 3H). ^13^C NMR (100 MHz, CDCl_3_) δ (ppm): 144.2, 141.1, 140.3, 136.0, 130.8, 130.8, 128.8, 123.6, 122.4, 121.2, 120.5, 109.7, 43.6, 41.6, 31.7, 29.3, 27.3, 22.7, 14.1.

#### 2.1.3. Synthesis of 14-hexyl-14*H*-bis[1]benzothieno[3,2-*b*:2’,3’-*h*]carbazole (**1a**)

**2b** (200 mg, 0.379 mmol) and phosphorus pentoxide (27 mg, 0.19 mmol) were dissolved in trifluoromethanesulfonic acid (5.7 mL) and stirred at room temperature for 72 h. The crude was poured into a mixture of ice-water (50 mL) and the resulting yellow precipitate was filtered. The precipitate was then dissolved in pyridine (30 mL) and refluxed overnight. The product was precipitated by adding water to the mixture, filtered and purified by flash column chromatography using a mixture of hexane and dichloromethane (10:1 *v*/*v*) as eluent. Compound **1a** was obtained as a bright yellow solid in a yield of 95% (166 mg, 0.358 mmol). ^1^H NMR (400 MHz, CDCl_3_) δ (ppm): 8.49 (s, 2H), 8.24–8.20 (m, 2H), 8.04 (s, 2H), 7.84–7.79 (m, 2H), 7.46–7.39 (m, 4H), 4.45 (t, *J* = 7.3 Hz, 2H), 2.01–1.92 (m, 2H), 1.44–1.19 (m, 6H), 0.82 (t, *J* = 7.2 Hz, 3H). ^13^C NMR (100 MHz, CDCl_3_) δ (ppm): 140.6, 140.5, 135.8, 134.7, 130.5, 126.8, 124.2, 123.4, 123.1, 121.5, 114.2, 100.5, 43.6, 31.8, 28.7, 27.2, 22.7, 14.2.

#### 2.1.4. Synthesis of 2,7-bis(4-hexylphenyl)-9-methyl-9*H*-carbazole (**5**)

2,7-Dibromo-9-methyl-9*H*-carbazole **4a** (1.00 g, 2.95 mmol), 4-hexylphenylboronic acid (1.58 g, 7.67 mmol), Pd(PPh_3_)_4_ (0.17 g, 0.15 mmol) and K_2_CO_3_ (2.44 g, 17.7 mmol) were dissolved in a mixture of THF and water (6:1 *v*/*v*, 62 mL) under nitrogen and stirred at reflux overnight. The reaction mixture was extracted with CH_2_Cl_2_, dried over MgSO_4_ and the solvent was removed under reduced pressure. The crude was purified by flash column chromatography using a mixture of hexane and ethyl acetate (100:1 *v*/*v*) as eluent. Compound **5** was obtained as a white solid in a yield of 65% (0.97 g, 1.93 mmol). ^1^H NMR (400 MHz, CDCl_3_) δ (ppm): 8.12 (d, *J* = 8.1 Hz, 2H), 7.66 (d, *J* = 7.9 Hz, 4H), 7.58 (d, *J* = 1.5 Hz, 2H), 7.48 (dd, *J* = 8.1, *J* = 1.5 Hz, 2H), 7.30 (d, *J* = 7.9 Hz, 4H), 3.93 (s, 3H), 2.68 (t, *J* = 7.9 Hz, 4H), 1.68 (m, 4H), 1.43–1.29 (m, 12H), 0.91 (t, *J* = 7.0 Hz, 6H).

#### 2.1.5. Synthesis of 3,6-dibromo-2,7-bis(4-hexylphenyl)-9-methyl-9*H*-carbazole (**6**)

**5** (0.968 g, 1.93 mmol) was dissolved in a mixture of glacial acid acetic and chloroform (1:1 *v*/*v*, 70 mL) at 0 °C. *N*-bromosuccinimide (0.704 g, 3.96 mmol) was added in portions and the mixture was stirred overnight at room temperature in the dark. The reaction was quenched with water and the product was extracted with CH_2_Cl_2_, dried over MgSO_4_ and the solvent was removed under reduced pressure. The crude was purified by flash column chromatography using hexane as eluent. Compound **6** was obtained as a white solid in a yield of 86% (1.09 g, 1.65 mmol). ^1^H NMR (400 MHz, CDCl_3_) δ (ppm): 8.32 (s, 2H), 7.43 (d, *J* = 8.1 Hz, 4H), 7.34 (s, 2H), 7.29 (m, *J* = 8.1 Hz, 4H), 3.78 (s, 3H), 2.69 (t, *J* = 7.8 Hz, 4H), 1.69 (m, 4H), 1.45–1.29 (m, 12H), 0.91 (t, *J* = 7.0 Hz, 6H).

#### 2.1.6. Synthesis of 2,7-bis(4-hexylphenyl)-9-methyl-3,6-bis(methylthio)-9*H*-carbazole (**3a**)

**6** (510 mg, 0.776 mmol) was dissolved in anhydrous THF (30 mL) under nitrogen and cooled to −78 °C. n-butyllithium (2.5 M in hexane, 0.78 mL, 1.9 mmol) was carefully added and stirred for 1 h. Then, dimethyl disulfide (0.17 mL, 1.9 mmol) was added dropwise, and the mixture was stirred at room temperature overnight. The crude was purified by flash column chromatography using a mixture of hexane and dichloromethane (10:1 *v*/*v*) as eluent. Compound **3a** was obtained as a white solid in a yield of 62% (287 mg, 0.484 mmol). ^1^H NMR (400 MHz, CDCl_3_) δ (ppm): 8.08 (s, 2H), 7.45 (d, *J* = 7.9 Hz, 4H), 7.28 (d, *J* = 7.9 Hz, 4H), 7.28 (s, 2H), 3.79 (s, 3H), 2.69 (t, *J* = 7.8 Hz, 4H), 2.41 (s, 6H), 1.69 (m, 4H), 1.45–1.29 (m, 12H), 0.91 (t, *J* = 6.9 Hz, 6H). ^13^C NMR (100 MHz, CDCl_3_) δ (ppm): 142.3, 140.9, 140.2, 139.0, 129.7, 128.2, 126.9, 122.0, 120.2, 110.7, 36.0, 31.9, 31.6, 29.4, 29.3, 22.8, 18.4, 14.3.

#### 2.1.7. Synthesis of 2,7-bis(4-hexylphenyl)-9-methyl-3,6-bis(methylsulfinyl)-9*H*-carbazole (**3b**)

**3a** (200 mg, 0.337 mmol) was dissolved in a mixture of glacial acetic acid and chloroform (1:1 *v*/*v*, 24 mL). Hydrogen peroxide (35%, 61 µL, 0.72 mmol) was subsequently added and the mixture was stirred overnight at room temperature. The solvents were removed under reduced pressure and the crude was purified by flash column chromatography using a mixture of hexane and ethyl acetate (1:1 *v*/*v*) as eluent. Compound **3b**, corresponding to a diastereomeric mixture, was obtained as a pale yellow solid in a yield of 86% (182 mg, 0.291 mmol). ^1^H NMR (400 MHz, CDCl_3_) δ (ppm): 8.96 and 8.95 (s and s‘, 2H), 7.41 and 7.40 (d and d‘, *J* = 8.0 Hz, 4H), 7.36 (s and s‘, 2H), 7.31 (d, *J* = 8.0 Hz, 4H), 3.91 (s, 3H), 2.70 (t, *J* = 7.8 Hz, 4H), 2.48 and 2.44 (s and s‘, 6H), 1.69 (m, 4H), 1.45–1.30 (m, 12H), 0.91 (t, *J* = 7.0 Hz, 6H). ^13^C NMR (100 MHz, CDCl_3_) δ (ppm): 143.4, 143.4, 143.0, 142.9, 138.4, 138.2, 136.0, 135.5, 135.5, 129.6, 129.5, 129.0, 128.9, 128.9, 122.5, 122.4, 117.3, 117.2, 110.7, 110.7, 42.7, 42.5, 35.8, 31.8, 31.5, 29.8, 29.1, 22.7, 14.2.

#### 2.1.8. Synthesis of 3,10-dihexyl-14-methyl-14*H*-bis[1]benzothieno[3,2-*b*:2’,3’-*h*]carbazole (**1b**)

**3b** (182 mg, 0.291 mmol) and phosphorus pentoxide (21 mg, 0.15 mmol) were dissolved in trifluoromethanesulfonic acid (4.4 mL) and stirred at room temperature for 72 h. The crude was poured into a mixture of ice-water (50 mL) and the resulting yellow precipitate was filtered. The precipitate was then dissolved in pyridine (25 mL) and refluxed overnight. The product was precipitated by adding water to the mixture, filtered and purified by flash column chromatography using a mixture of hexane and dichloromethane (10:1 *v*/*v*) as eluent. Compound **1b** was obtained as a dark yellow solid in a yield of 66% (107 mg, 0.190 mmol). ^1^H NMR (400 MHz, CDCl_3_) δ (ppm): 8.50 (s, 2H), 8.16 (d, *J* = 8.1 Hz, 2H), 8.04 (s, 2H), 7.68 (s, 2H), 7.31 (dd, *J* = 8.1, *J* = 1.5 Hz, 2H), 4.03 (s, 3H), 2.78 (t, *J* = 7.8 Hz, 4H), 1.73 (m, 4H), 1.45–1.27 (m, 12H), 0.90 (t, *J* = 7.1 Hz, 6H). ^13^C NMR (125 MHz, CDCl_3_) δ (ppm): 142.1, 141.2, 140.6, 134.8, 133.7, 130.5, 125.2, 122.9, 122.5, 121.2, 114.0, 100.0, 36.7, 31.9, 31.8, 29.2, 22.8, 14.3.

#### 2.1.9. Synthesis of 3,6-bis(2-methylthiophenyl)-9-hexyl-9*H*-carbazole (**7a**)

3,6-dibromo-9-hexyl-9*H*-carbazole (1.15 g, 2.81 mmol), 2-methylthiophenylboronic acid (1.23 g, 7.31 mmol), Pd(PPh_3_)_4_ (0.162 g, 0.140 mmol) and K_2_CO_3_ (2.33 g, 16.9 mmol) were dissolved in a mixture of THF and water (6:1 *v*/*v*, 58 mL) under nitrogen and stirred at reflux overnight. The reaction mixture was extracted with CH_2_Cl_2_, dried over MgSO_4_ and the solvent was removed under reduced pressure. The crude was purified by flash column chromatography using a mixture of hexane and dichloromethane (20:1 *v*/*v*) as eluent. Compound **7a** was obtained as a white solid in a yield of 85% (1.190 g, 2.400 mmol). ^1^H NMR (400 MHz, CDCl_3_) δ (ppm): 8.27 (dd, *J* = 8.0, 1.5 Hz, 2H), 8.18 (d, *J* = 1.5 Hz, 2H), 7.69–7.63 (m, 4H), 7.59–7.54 (m, 2H), 7.51 (d, *J* = 8.0 Hz, 4H), 7.48 (dd, *J* = 7.5, 1.3 Hz, 4H), 4.38 (t, *J* = 7.4 Hz, 2H), 2.59 (s, 6H), 2.00–1.91 (m, 2H), 1.50–1.30 (m, 6H), 0.90 (t, *J* = 7.1 Hz, 3H). ^13^C NMR (100 MHz, CDCl_3_) δ (ppm): 141.8, 140.4, 137.7, 131.4, 130.7, 127.6, 127.4, 125.1, 124.8, 123.0, 121.4, 108.4, 43.6, 31.8, 29.2, 27.2, 22.7, 16.1, 14.2.

#### 2.1.10. Synthesis of 3,6-bis(2-methylsulfinylphenyl)-9-hexyl-9*H*-carbazole (**7b**)

**7a** (0.250 g, 0.504 mmol) was dissolved in glacial acetic acid (17 mL) and cooled at 0 °C. Then, hydrogen peroxide (35%, 93 µL, 1.1 mmol) was added carefully and the mixture was stirred overnight at room temperature. The solvents were removed under reduced pressure. The crude was purified by flash column chromatography using a mixture of hexane and ethyl acetate (1:1 *v*/*v*) as eluent. Compound **7b**, corresponding to a diastereomeric mixture, was obtained as a pale yellow solid in a yield of 95% (0.254 g, 0.481 mmol). ^1^H NMR (400 MHz, CDCl_3_) δ (ppm): 8.17–8.13 (m, 2H), 8.11–8.07 (m, 2H), 7.65–7.55 (m, 4H), 7.55–7.48 (m, 4H), 7.45 and 7.42 (dd + dd’, *J* = 7.4, 1.3 Hz, 2H), 4.38 (t, *J* = 7.4 Hz, 2H), 2.37 and 2.35 (s + s’, 6H), 2.00–1.91 (m, 2H), 1.53–1.29 (m, 6H), 0.90 (t, *J* = 7.1 Hz, 3H). ^13^C NMR (100 MHz, CDCl_3_) δ (ppm): 144.3, 140.7, 140.2, 131.1, 131.0, 130.9, 130.8, 129.2, 128.5, 127.5, 123.6, 123.0, 121.3, 121.3, 109.3, 43.7, 41.7, 41.7, 31.7, 29.2, 27.2, 22.72, 14.1.

### 2.2. Instrumentation and Methods

^1^H NMR (400 MHz) and ^13^C NMR (100 MHz) spectra were acquired in a Varian Mercury spectrometer. ^13^C NMR (125 MHz) spectra were acquired in a Bruker spectrometer (Bremen, Germany). NMR spectra were processed using the MestRec Nova software (version 14.2.0) and referenced with the solvent signal. Absorption and emission spectra were recorded in a Varian Cary UV-Vis-NIR 500E spectrophotometer (Palo Alto, CA, USA) and a PTI fluorimeter (Birmingham, AL, USA), respectively. Fluorescence quantum yields (Φ_f_) were calculated using a reported protocol [32]. For this, 1,4-bis(5-phenyl-2-oxazolyl)benzene (POPOP) was employed as standard, possessing a Φ_f_ = 0.93 in cyclohexane after excitation at 300 nm. Cyclic voltammograms were collected in a cylindrical three-electrode cell using a microcomputer-controlled potentiostat/galvanostat Autolab with PGSTAT30 equipment and GPES software (version 4.9). An Ag/Ag^+^ electrode (10^−3^ M AgNO_3_ in acetonitrile) was used as the reference electrode. The working and counter electrodes consisted of a glassy-carbon electrode and a platinum wire, respectively. All voltammograms were acquired under quiescent conditions and under argon atmosphere at 100 mV s^−1^. All compounds were dissolved in distilled dichloromethane (10^−3^ M) and the supporting electrolyte employed was tetrabutylammonium hexafluorophosphate (TBAP, 0.1 M). All potentials were referred to the Fc^+^/Fc redox couple. The ionization potentials (IP) were estimated from the onset of the first oxidation peak (^ox^E_onset_) as IP = ^ox^E_onset_ + 5.39, where 5.39 eV corresponds to the formal potential in the Fermi scale of the Fc^+^/Fc couple [33]. The electron affinities (EA) were estimated as EA = IP – E_gap_. The optical gap energies (E_gap_) were obtained from the λ_onset_ of the absorption spectra. The thermogravimetric analyses (TGA) and the differential scanning calorimetry (DSC) thermograms were obtained under nitrogen atmosphere in a TA Instruments Q50 (New Castle, DE, USA) and TA Instruments Q2000 calorimeter, respectively. The scan rates were 20 °C min^−1^ for the TGA and 10 °C min^−1^ for the DSC. The single-crystal elucidation was achieved on a D8 Venture System (Bruker AXS, Karlsruhe, Germany), equipped with a multilayer monochromator and a Mo microfocus (λ = 0.71073 Å). The frames were integrated using the Bruker SAINT software package (version SAINT V8.38A) with a narrow-frame algorithm. The structure resolution and refinement were performed using the Bruker SHELXTL software package (version APEX v2018 7-2). Out-of-plane GIXRD measurements of thin-films (75 nm thickness) were collected in a PANalytical X’Pert PRO MRD diffractometer (Almelo, the Netherlands). It consisted of a PIXcel detector, a parabolic Göbel mirror at the incident beam and a parallel plate collimator at the diffracted beam, with Cu Kα radiation (λ = 1.5418 Å) and a work power of 45 kV × 40 mA. An optimized angle of incidence around 0.17° was used for the measurements.

### 2.3. OTFT Fabrication and Characterization

The fabricated OTFTs were based on the bottom-gate top-contact architecture. The substrates consisted of thermally oxidized crystalline silicon wafers with gate dielectric (SiO_2_) thicknesses ranging from 125 to 135 nm. The gate side of the silicon wafer was partially treated with ammonium fluoride. The substrates were then cleaned by subsequent ultrasonic treatments in acetone, 2-propanol and water, dried using a nitrogen blow and heated at 100 °C for 5 min. The SiO_2_ surface was covered with either polystyrene (PS) or octadecyltrichlorosilane (OTS) as an organic dielectric. The deposition of the PS layer was carried out using a solution of PS in toluene (4 mg mL^−1^), which was spin-coated onto the wafer. The substrate was spun at 1500 rpm for 5 s and 2500 rpm for 33 s employing a P6700 spin-coater. The substrates were then annealed at 120 °C for 1 h. The formation of the OTS Self Assembled Monolayer (SAM) [34,35] was achieved by immersing the substrates in a solution of OTS (2 mM) in toluene for 24 h at room temperature. The substrates were cleansed by subsequent ultrasonic treatments in toluene, acetone and 2-propanol, and eventually dried using a nitrogen blow and heated at 100 °C for 5 min. The organic compounds were deposited by thermal evaporation under vacuum in a chamber with a pressure below 10^−6^ mbar. The sublimation temperature for each compound was controlled manually to maintain the deposition at a stable rate of 0.3 Å s^−1^ until the semiconductor layers of about 75 nm thickness were obtained. The wafers were subsequently transferred to another vacuum system to deposit the golden contacts. The drain and source electrodes were defined by a metallic mask, providing a channel length and width of 80 µm and 2 mm, respectively. The resultant OTFTs were characterized under ambient conditions in the dark. The electrical characteristics were recorded employing a Keithley 2636A source meter (Solon, OH, USA). The charge carrier mobility was calculated in the saturation regime (*µ_sat_*) from Equation (1):

(1) $$I_{D}=\frac{W\;C_{\mathrm{ox}}\;\mu }{2\;L}{\left(V_{G}{-V}_{\mathrm{th}}\right)}^{2}$$

where *W* and *L* correspond to the channel width and length, respectively, and *C*_ox_ is the capacitance per unit area of the gate insulator.

## 3. Results and Discussion

### 3.1. Synthesis

**Figure 2:**
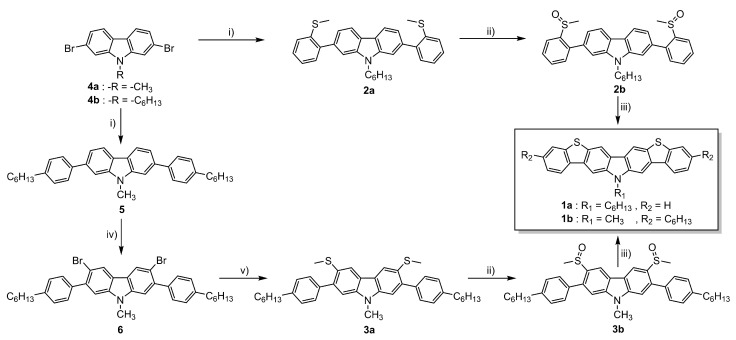
Synthetic routes followed to obtain the final compounds **1a**–**b**. Reagents and conditions: (i) Boronic acid (4-hexylphenylboronic acid for **5** and 2-methylthiophenylboronic acid for **2a**), Pd(PPh_3_)_4_, K_2_CO_3_ in THF:H_2_O, reflux. (ii) H_2_O_2_ in AcOH (**2b**) or AcOH:CHCl_3_ (**3b**), RT. (iii) a. CF_3_SO_3_H, P_2_O_5_, RT; b. pyridine, reflux. (iv) NBS in AcOH:CHCl_3_, RT. (v) a. BuLi in THF, −78 °C; b. CH_3_SSCH_3_, RT.

The synthetic routes that led to the final compounds **1a**–**b** are presented in Figure 2.

The starting materials **4a**–**b** were prepared by alkylating 2,7-dibromo-9*H*-carbazole, which was synthesized as described in the literature [36,37], under standard conditions [22]. Compound **2a** was obtained from **4b** through the Suzuki–Miyaura cross-coupling reaction [38] with the 2-methylthiophenylboronic acid and it was subsequently oxidized to the corresponding sulfoxide **2b** with H_2_O_2_ in glacial acetic acid. Finally, the corresponding cyclization with triflic acid followed by the demethylation in pyridine furnished compound **1a** [25].

The synthesis of **1b** was performed following a reported method [25]. It consisted of the attachment of the 4-hexylphenylboronic acid to the methylated 2,7-dibromo-9*H*-carbazole **4a** through the Suzuki–Miyaura cross-coupling reaction followed by the dibromination of positions 3 and 6 of the carbazole ring. Then, the substitution of the bromines with dimethyl disulfide led to compound **3a**. After the oxidation to **3b**, the consecutive cyclization provided compound **1b**.

### 3.2. Optical, Electrochemical and Thermal Properties

#### 3.2.1. Sulfurated Carbazole Precursors

In view of the interesting and diverse optical properties of the sulfurated precursors implied in the synthesis of **1a**–**b**, we decided to further investigate the relationship between emission and structural design. Both the emitted color and the quantum yield of the studied fluorophores proved to be strongly conditioned by the oxidation degree of the sulfur moieties and their bonding positions with respect to the carbazole ring. As an additional comparison, we synthesized the complementary derivatives **7a**–**b** analogous to **2a**–**b**, in which the 2-methylthiophenyl moiety is attached to the carbazole ring at positions 3 and 6 instead. Synthetically, they were prepared using the same conditions employed for **2a**–**b**, and starting from the commercially available 3,6-dibromo-9*H*-carbazole. The photoluminescence data of all the aforementioned fluorophores is compiled in Table 1.

**Figure 3:**
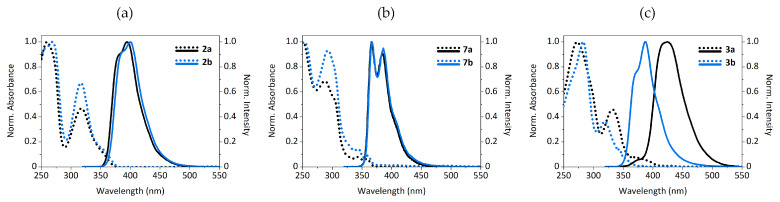
Absorption (pointed) and emission (solid, λ_ex_ = 300 nm) spectra in CH_2_Cl_2_ of compounds: (**a**) **2a**–**b**; (**b**) **7a**–**b**; (**c**) **3a**–**b**.

All derivatives were analyzed in three different solvents with increasing polarity, namely, cyclohexane, dichloromethane and acetonitrile. As observed, the optical properties on the tested solvents provide similar results in terms of the quantum yields and an expected slight bathochromic shift from cyclohexane to the other two solvents (the corresponding spectra are shown in Figure S1). Hence, the subsequent discussion is focused on the results obtained in dichloromethane as representatives. The absorption and emission spectra in this solvent are shown in Figure 3.

The derivatives containing the 2-methylthiophenyl moiety (**2a** and **7a**) display similar emission spectra with respect to their oxidized counterparts **2b** and **7b**. In the case of **2a** and **2b**, the former peaks at 394 nm, while the latter shows a very slight bathochromic shift, peaking at 400 nm. Concerning, compounds **7a** and **7b**, the shift is even smaller. However, the oxidation state of the sulfur in structure **3** is more determining. Contrary to **2** and **7**, the oxidation process causes a remarkable hypsochromic shift of 37 nm, from 424 to 387 nm for **3a** and **3b**, respectively.

On the other hand, the fluorescence quantum yields of both sulfur and sulfoxide derivatives provided unexpected results. Several reported methylsulfinyl-containing fluorophores display a much lower quantum yield than their methylthio analogues [39], and this abrupt change of the emission has found applications in fields such as biological imaging and detection probes [30,31]. Nevertheless, in this case we found the opposite trend, i.e., the oxidation to sulfoxide leads to a noteworthy enhancement of the quantum yield with respect to the almost non-emitting sulfur derivatives. Even though there are some examples that follow this tendency [40,41], the difference between the quantum yields of both counterparts is seldom as significant as the ones herein reported. Specifically, compounds **2a** and **7a**, featuring the 2-methylthiophenyl unit, display low quantum yields of 0.03 and 0.05, respectively, whereas those of their oxidized analogues **2b** and **7b** increase to 0.34 and 0.12, respectively. The increase observed in the *meta*-substituted carbazole **2** is considerably higher than in the *para*-substituted derivative **7**. Furthermore, the first one has a larger contribution in the blue region of the visible spectrum. In the case of compounds **3a**–**b**, the difference is even more remarkable, with a 16-fold enhancement between analogues. The fact that the methylthio group is directly bounded to the carbazole heterocycle in compound **3** seemingly makes it more susceptible to the changes of the oxidation state of the sulfur. Furthermore, considering the hypsochromic shift associated with the oxidation process, compounds **3a**–**b** appear as a promising switchable redox couple in terms of optical applications. As a whole, the combination of the carbazole ring with the methylthio moiety leads to a wide assortment of fluorophores with distinct optical properties that can be finely tuned through the structural design.

#### 3.2.2. Bisbenzothienocarbazole Derivatives

The thermal, optical and electrochemical characteristics of the bisbenzothienocarbazole derivatives **1a**–**b** are detailed in Table 2. This characterization mainly focuses on determining whether these compounds display suitable properties to be applied in OTFTs, i.e., high thermal stability and appropriate energy levels.

**Figure 4:**
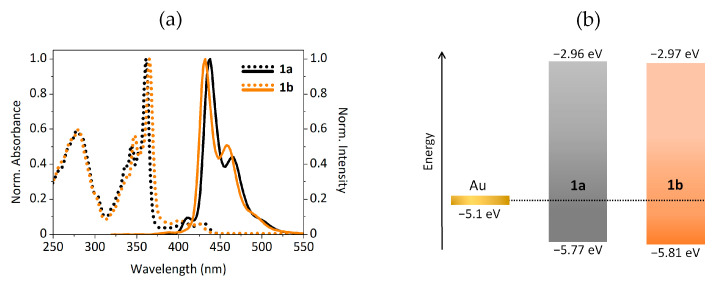
(**a**) Absorption (dotted) and emission (solid, λ_ex_ = 300 nm) spectra of compounds **1a**–**b**. (**b**) Energy levels of compounds **1a**–**b** with respect to the gold electrodes.

Both compounds exhibited excellent thermal stability, necessary for the vacuum deposition process during the OTFT fabrication, with onset decomposition temperatures (T_d_) higher than 400 °C. The emission of the bisbenzothienocarbazole derivatives, measured in CH_2_Cl_2_, have an expected bathochromic shift in comparison with their precursors derived from the extension of the aromatic core (e.g., 38 nm from **2b** to **1a**, peaking at 400 and 438 nm, respectively). Furthermore, the fluorescence quantum yields diminish regarding the values displayed by their methylsulfinyl precursors, but still outpace their methylthio equivalents. Considering their energy levels, compounds **1a**–**b** possess similar ionization potential (IP) values, calculated from the first oxidation onset potential, and optical gap energy values, estimated from their absorption spectra (shown in Figure 4a). The resulting energy levels, represented in Figure 4b, have estimated values around −5.80 and −2.97 eV for their HOMO and LUMO energy levels, respectively. Their low-lying HOMO energy levels, which confer more stability against oxidation by atmospheric oxygen [4,42], are also suitable for the gold work function (i.e., 5.1 eV), making this set of bisbenzothienocarbazole derivatives appropriate for the integration in hole transporting OTFTs.

### 3.3. Organic Thin-Film Transistors

The hole transporting properties of the bisbenzothienocarbazole derivatives **1a**–**b** were studied in standard bottom gate-top contact OTFTs. The organic compounds were deposited as the active layers (75 nm of thickness) by vacuum evaporation, as well as the gold for the source and drain electrodes. The SiO_2_ dielectric surface was treated with either octadecyltrichlorosilane (OTS) or polystyrene (PS). Table 3 represents the maximum and average hole mobility values (*µ_h,max_* and *µ_h,avg_,* respectively) as well as the *I_on_*/*I_off_* ratio for both compounds in each SiO_2_ surface treatment.

**Figure 5:**
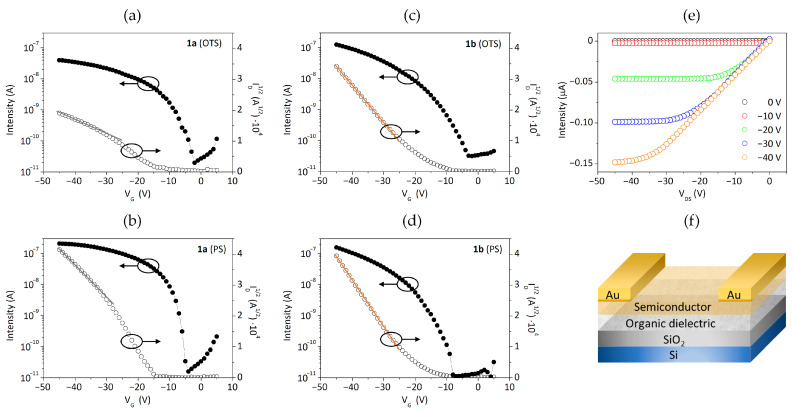
OTFT characteristics of representative devices fabricated with compounds **1a**–**b**: transfer (V_DS_ = −40 V) and saturation characteristics with the linear adjustment employed to extract *µ_h_* of compound **1a** in (**a**) OTS- and (**b**) PS-treated devices; of compound **1b** in (**c**) OTS- and (**d**) PS-treated devices; (**e**) output characteristics of a PS-treated device fabricated with compound **1a**; (**f**) scheme of the studied bottom gate-top contact OTFTs.

Both compounds displayed p-type behavior, with the best performance found in devices integrating compound **1b**, with mobility values up to 1.1 × 10^−3^ cm^2^ V^−1^ s^−1^ in PS-treated devices. In the case of compound **1a**, the highest mobility was also found in PS-treated devices, with values up to 4.4 × 10^−4^ cm^2^ V^−1^ s^−1^, whereas the mobility drops to 7.1 × 10^−5^ cm^2^ V^−1^ s^−1^ in devices containing OTS. The effect of the chosen dielectric in this derivative is therefore more significant, with a nearly 6-fold improvement deposited over PS with respect to OTS. Seemingly, the presence of the peripheral alkyl chains in **1b** endows the core with more adaptability towards both surface treatments regardless of their nature. Notably, all devices showed noteworthy *I_on_*/*I_off_* ratios ranging from 10^3^ to 10^4^, being generally superior in devices based on PS as dielectric. This fact correlates with the outperformance of both bisbenzothienocarbazole derivatives over PS. The OTFT characteristics of a representative set of devices and the utilized OTFT architecture are represented in Figure 5.

Another aspect that should be mentioned is that all devices fabricated from derivatives **1a**–**b** tend to exhibit a non-ideal behavior, as observed by the different nonlinearities present in the OTFT saturation characteristics. Devices based on derivative **1a** generally reveal a kink in the saturation characteristics (Figure 5a,b), in which the slope at high V_G_ is lower and higher at low V_G_. The mobility values were extracted from the high V_G_ region, accordingly to what is suggested in the literature [43,44]. Concerning compound **1b**, the devices show more linear characteristics but still with a significant threshold voltage (Figure 5c,d), so the resultant charge mobility was extracted consequently [43].

All devices were measured and stored under ambient conditions in the dark. Remarkably, devices based on compound **1a** deposited over PS featured the longest lifetime, maintaining a charge mobility up to 2 × 10^−4^ cm^2^ V^−1^ s^−1^ after seven months. The corresponding transfer and output characteristics after fabrication are shown in Figure 5b,e, whereas the equivalent ones obtained after seven months can be found in Figure S2. This fact also supports the high potential of the bisbenzothienocarbazole core.

### 3.4. Crystallographic Data and X-ray Analysis

In view of the dependence of the device performance with the alkylation patterning of the studied core, we decided to further analyze its role in the molecular arrangement and degree of order of the deposited films. With this aim, the single crystal structures of compounds **1a**–**b** as well as the GIXRD patterns of the films were investigated.

**Figure 6:**
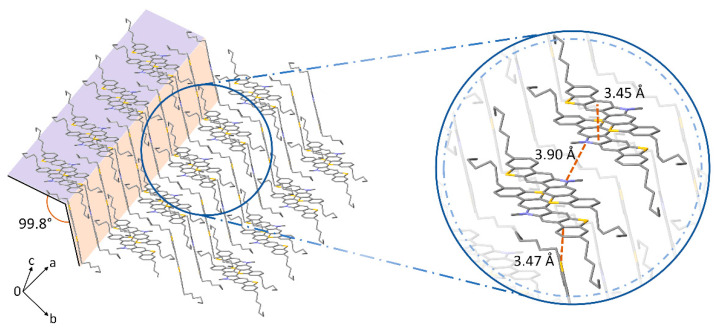
Single crystal structure of compound **1b**, with the corresponding interplanar angle. The amplified region shows the π-π stacking distance (3.45 Å), the N···N distance (3.90 Å) between dimers and the edge-to-face S···π interaction (3.47 Å). Hydrogens have been excluded for clarity.

Compound **1b** crystallized in space group P_−1_ of the triclinic system (Figure 6, Table S1 in the Supplementary Materials). The corresponding crystal structure reveals differentiated dimers of co-facial molecules disposed in a sandwich herringbone packing. The pair of molecules involved are separated by 3.45 Å through π-π stacking interactions, affording a significant π-orbital overlap. Regarding the intermolecular packing between dimers, it exhibits a slightly slipped disposition. In fact, the separation between nitrogens displays the shortest π-π distance found between dimers, e.g., 3.90 Å. This slipped disposition is mostly assisted by tilted edge-to-face C-H···π and S···π interactions, as well as the additional interactions from the peripheral alkyl chains. As a result, the interplanar angle is almost perpendicular, which along the slipped disposition associated with the packing could hamper an efficient π-π charge hopping [45].

**Figure 7:**
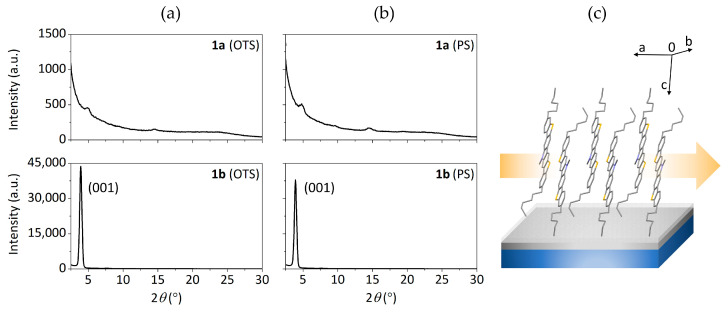
GIXRD patterns of vacuum-deposited films of compounds **1a**–**b** on: (**a**) OTS- and (**b**) PS-treated Si/SiO_2_ substrates; (**c**) proposed molecular disposition of **1b** in the film based on the reflections observed in the GIXRD patterns, with the (001) plane situated parallel to the substrate and the arrow indicating the resulting π-π stacking direction. Hydrogens have been excluded for clarity.

Compound **1a** was reported to crystallize in space group P2_1/n_ of the orthorhombic system in the presence of CHCl_3_ [25]. In spite of the efforts invested in producing solvent-free crystals proper for measuring, it was not possible in any of the alternative solvents tested. Seemingly, compound **1a** depends upon small solvent molecules such as CHCl_3_ or CH_2_Cl_2_ to stabilize the packing, affecting the intermolecular arrangement. This is also evident by analyzing the X-ray diffraction patterns of the deposited films. Considering the literature [25], the XRD pattern of a **1a** drop-casted thin film provides sharp and intense diffraction peaks typical of crystalline layers with a high degree of order. However, the herein performed GIXRD measurements of **1a** vacuum-deposited films over Si/SiO_2_ substrates (Figure 7) reveal generally amorphous layers with very small diffraction peaks at 2*θ* = 4.9 and 14.5°. The diffraction peaks are subtly more intense in the PS-treated substrate, confirming a higher degree of order in the outperforming PS-based devices. Overall, the obtained results support the theory that the presence of small solvent units are necessary within the intermolecular arrangement to achieve highly ordered films, preventing a superior performance in vacuum-deposited OTFTs. Taking this into account, compound **1a** appears as an interesting candidate for solution-processed devices.

On the other hand, GIXRD measurements of **1b** films exhibit a much higher degree of order than those based on **1a** in both dielectric treatments. The strong diffraction peak at 2*θ* = 3.9° was proposed to belong to the reflection 001 based on the powder pattern diffractogram and the one simulated from the single crystal structure. This implies that the π-π stacking direction lies parallel to the substrate, facilitating the charge transport. The GIXRD patterns and the proposed disposition over the substrate are shown in Figure 7. The overall better performance of **1b**-based devices can be explained by the higher degree of order present in the semiconductor layer. In the case of **1b**, the diffraction peak is slightly more intense in the OTS-treated surface, which in this case does not translate into a higher charge mobility in the respective device in comparison with the ones integrating PS.

As a whole, the alkylation patterning of the bisbenzothienocarbazole core exerts a significant effect over its charge transport properties, conditioning both the intermolecular disposition and the degree of order of the film. Indeed, the addition of hexyl chains on the peripheral sides of **1b** in a head-tail style derives into a better performance than the lateral *N*-hexyl substitution of **1a**. In terms of the device architecture, the inclusion of PS as the dielectric interlayer proved to be a convenient option to improve the charge mobility. Taking into account these two key factors, the performance of the bisbenzothienocarbazole core is susceptible to further optimization, confirming once again the potential of this core as an air stable semiconductor.

## 4. Conclusions

The two synthesized derivatives of the bisbenzothienocarbazole core, displaying high thermal stability and appropriate energy levels, have been integrated in OTFTs. The different alkylation patterning of the derivatives combined with the chosen dielectrics were revealed as key factors that conditioned the device performance. Specifically, the elongated architecture of derivative **1b** afforded a balanced performance both in OTS- and PS-treated devices, with the latter reaching the highest charge mobility value, i.e., 1.1 × 10^−3^ cm^2^ V^−1^ s^−1^. The role of the alkylation patterning at conferring a proper intermolecular arrangement was investigated by means of X-ray analysis. Derivative **1b** provides an intermolecular arrangement suitable for the charge transport and films with a high degree of order, which are in accordance with the overall better performance in OTFTs. Notably, the bisbenzothienocarbazole core provides OTFTs with substantial air stability, which in the case of derivative **1a** extends up to seven months. Additionally, their sulfur-containing synthetic precursors provided appealing results in terms of their optical properties. Interestingly, the chromophores bearing the methylsulfinyl moiety displayed a much higher quantum yield than their reduced counterparts. Thus, the resorted synthetic route resulted in a set of compounds with assorted properties and potential within diverse fields.

## Figures and Tables

**Table 1:** Photoluminescent characteristics of the sulfurated molecular structures **2**, **3** and **7** in different solvents.

Structure	Solvent	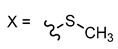			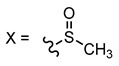		
		Comp.	λ_Em_	Φ_f_ ^1^	Comp.	λ_Em_	Φ_f_ ^1^
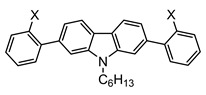	Cyclohexane	**2a**	384	0.07	**2b**	377, 388	0.33
	CH_2_Cl_2_		394	0.03		400	0.34
	CH_3_CN		393	0.04		400	0.35
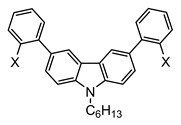	Cyclohexane	**7a**	360, 379	0.05	**7b**	363, 382	0.15
	CH_2_Cl_2_		366, 384	0.05		367, 386	0.12
	CH_3_CN		366, 383	0.05		367, 386	0.13
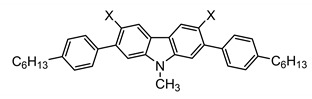	Cyclohexane	**3a**	406, 420	0.02	**3b**	370, 384	0.17
	CH_2_Cl_2_		424	0.01		387	0.16
	CH_3_CN		422, 431	0.01		374, 390	0.15

^1^ Fluorescent quantum yields (Φ_f_) were determined using POPOP as standard (λ_Ex_ = 300 nm).

**Table 2:** Thermal, optical and electrochemical properties of compounds **1a**–**b**.

Compound	T_m_ (°C) ^1^	T_d_ (°C) ^1^	λ_Abs_ ^2^	λ_Em_ ^2^	Φ_f_ ^2^	E_gap_ (eV) ^3^	^ox^E_onset_ (V) ^4^	IP (eV) ^5^	EA (eV) ^6^
**1a**	197	435	344, 361	438, 466	0.04	2.81	0.38	5.77	2.96
**1b**	217	443	347, 365	432, 458	0.05	2.84	0.42	5.81	2.97

^1^ Melting (T_m_) and onset decomposition (T_d_) temperatures. ^2^ Recorded in CH_2_Cl_2_ (10 µM). Fluorescent quantum yields (Φ_f_) were determined using POPOP as standard (λ_Ex_ = 300 nm). ^3^ Optical gap energies, estimated from the absorption spectrum. ^4^ Onset oxidation potentials, determinated from cyclic voltammetry in CH_2_Cl_2_ (1 mM). ^5^ Ionization potentials calculated as IP = ^ox^E_onset_ + 5.39. ^6^ Electron affinities calculated as EA = IP − E_gap_.

**Table 3:** Organic thin-film transistor (OTFT) characteristics of devices fabricated with compounds **1a**–**b** in octadecyltrichlorosilane (OTS)- and polystyrene (PS)-treated substrates.

Compound	Dielectric	*µ_h,max_* (cm^2^ V^−1^ s^−1^) ^1^	*µ_h,avg_* (cm^2^ V^−1^ s^−1^) ^2^	*I_on_*/*I_off_*
**1a**	OTS	7.1 × 10^−5^	(6.7 ± 0.5) × 10^−5^	~10^3^
	PS	4.4 × 10^−4^	(3.7 ± 0.6) × 10^−4^	~10^4^
**1b**	OTS	6.5 × 10^−4^	(5.5 ± 0.7) × 10^−4^	~10^3^
	PS	1.1 × 10^−3^	(1.0 ± 0.1) × 10^−3^	~10^4^

^1^ Maximum hole mobility values (*µ_h,max_*) extracted from the saturation regime. ^2^ Average hole mobility values (*µ_h,avg_*) calculated from eight representative devices.

## Data Availability

Data is contained within the article or Supplementary Materials.

## Supplementary Materials

The following are available online at https://www.mdpi.com/article/10.3390/ma14133487/s1, Figure S1: Emission spectra in cyclohexane (CH), dichloromethane (DCM) and acetonitrile (ACN) of compounds **2a** (a), **2b** (d), **7a** (c), **7b** (d), **3a** (e), **3b** (f), Figure S2: OTFT characteristics of a PS-treated device integrating compound **1a** seven months after fabrication: (a) transfer and (b) output characteristics, Table S1: Crystal data and structure refinement for compound **1b**.
