# Supplementary material for: Towards the Bisbenzothienocarbazole Core: A Route of Sulfurated Carbazole Derivatives with Assorted Optoelectronic Properties and Applications
Source: Materials (Basel). 2021 Jun 23;14(13):3487. doi: 10.3390/ma14133487 (PMC8269540; doi:10.3390/ma14133487)
Supplement: Supplementary file 1 [file materials-14-03487-s001.zip › materials-1255798-supplementary.pdf]

materials MDPI

Supplementary Materials

Towards the Bisbenzothienocarbazole Core: A Route of Sulfurated Carbazole Derivatives with Assorted Optoelectronic Properties and Applications

Roger Bujaldón [1], Joaquim Puigdollers [2] and Dolores Velasco [1,*]

[1] *Grup de Materials Orgànics, Institut de Nanociència i Nanotecnologia (IN²UB), Departament de Química Inorgànica i Orgànica, Secció de Química Orgànica, Universitat de Barcelona, Martí i Franquès, 1, E-08028, Barcelona, Spain.*

[2] *Departament d'Enginyeria Electrònica, Universitat Politècnica de Catalunya, Jordi Girona, 1-3, E-08034, Barcelona, Spain*

E-mail: dvelasco@ub.edu

• **Figure S1** S2

• **Figure S2** S2

• **Table S1** S3

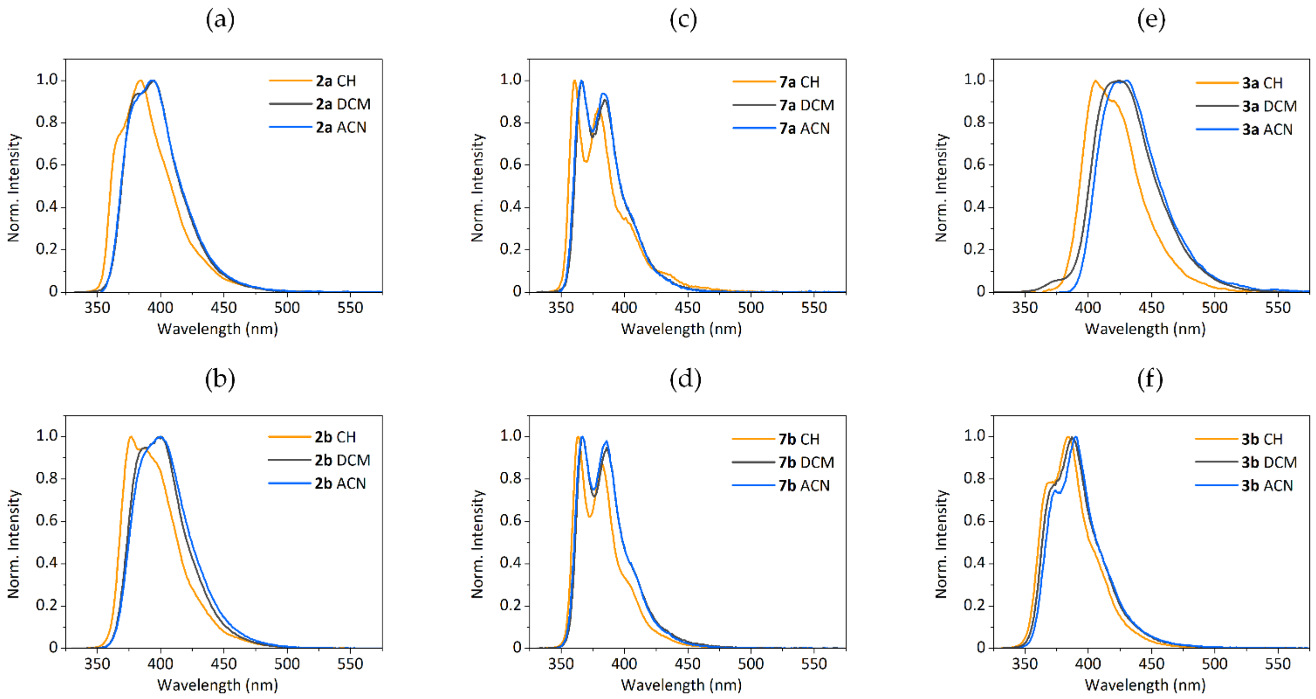

Figure S1. Emission spectra in cyclohexane (CH), dichloromethane (DCM) and acetonitrile (ACN) of compounds **2a** (a), **2b** (b), **7a** (c), **7b** (d), **3a** (e), **3b** (f).

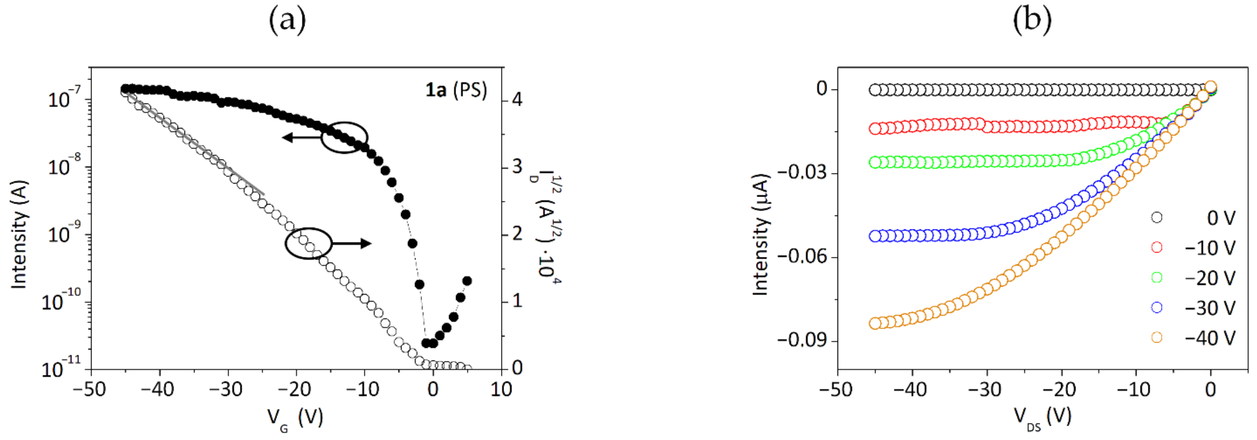

Figure S2. OTFT characteristics of a PS-treated device integrating compound **1a** seven months after the fabrication: (a) transfer and (b) output characteristics.

Table S1. Crystal data and structure refinement for compound **1b**.

	**1b**	
Identification code	Q38YB12_0m_a	
Empirical formula	C37 H39 N S2	
Formula weight	561.81	
Temperature	100(2) K	
Wavelength	0.71073 Å	
Crystal system	Triclinic	
Space group	P -1	
Unit cell dimensions	a = 8.4813(11) Å	α= 109.324(5)°.
	b = 18.194(2) Å	β= 90.767(6)°.
	c = 20.249(3) Å	γ = 90.378(5)°.
Volume	2948.1(7) Å^3	
Z	4	
Density (calculated)	1.266 Mg/m^3	
Absorption coefficient	0.208 mm^{-1}	
F(000)	1200	
Crystal size	0.468 x 0.165 x 0.083 mm^3	
Theta range for data collection	2.132 to 33.428°.	
Index ranges	-13<=h<=13, -27<=k<=28, -31<=l<=31	
Reflections collected	110760	
Independent reflections	22561 [R(int) = 0.1591]	
Completeness to theta = 25.242°	99.9 %	
Absorption correction	Semi-empirical from equivalents	
Max. and min. transmission	0.7465 and 0.6196	
Refinement method	Full-matrix least-squares on F^2	
Data / restraints / parameters	22561 / 0 / 728	
Goodness-of-fit on F^2	1.017	
Final R indices [I>2sigma(I)]	R1 = 0.1005, wR2 = 0.2491	
R indices (all data)	R1 = 0.1747, wR2 = 0.3059	
Extinction coefficient	n/a	
Largest diff. peak and hole	1.137 and -0.933 e.Å^{-3}	
